# Wheat growth responses to soil mechanical impedance are dependent on phosphorus supply

**DOI:** 10.1016/j.still.2020.104754

**Published:** 2021-01

**Authors:** Xin Wang, Jianbo Shen, Peter Hedden, Andrew L. Phillips, Stephen G. Thomas, Yaoxiang Ge, Rhys W. Ashton, William R. Whalley

**Affiliations:** a Department of Plant Nutrition, College of Resources and Environmental Sciences, Key Laboratory of Plant-Soil Interactions, MoE, National Academy of Agriculture Green Development, China Agricultural University, Beijing, 100193, PR China; b Rothamsted Research, West Common, Harpenden, AL5 2JQ, UK; c Laboratory of Growth Regulators, Czech Academy of Sciences, Institute of Experimental Botany & Palacký University, Šlechtitelů 27, CZ-78371 Olomouc, Czech Republic; d College of Biology and Pharmacy, Yulin Normal University, Yulin, 537000, PR China

**Keywords:** Root impedance, Phosphorus absorption, *Triticum aestivum*, Rht-1 dwarfing alleles, Gibberellin sensitivity

## Abstract

Increased mechanical impedance induced by soil drying or compaction causes reduction in plant growth and crop yield. However, how mechanical impedance interacts with nutrient stress has been largely unknown. Here, we investigated the effect of mechanical impedance on the growth of wheat seedlings under contrasting phosphorus (P) supply in a sand culture system which allows the mechanical impedance to be independent of water and nutrient availability. Two wheat genotypes containing the *Rht-B1a* (tall) or *Rht-B1c* (gibberellin-insensitive dwarf) alleles in the Cadenza background were used and their shoot and root traits were determined. Mechanical impedance caused a significant reduction in plant growth under sufficient P supply, including reduced shoot and root biomass, leaf area and total root length. By contrast, under low P supply, mechanical impedance did not affect biomass, tiller number, leaf length, and nodal root number in both wheat genotypes, indicating that the magnitude of the growth restriction imposed by mechanical impedance was dependent on P supply. The interaction effect between mechanical impedance and P level was significant on most plant traits except for axial and lateral root length, suggesting an evident physical and nutritional interaction. Our findings provide valuable insights into the integrated effects of plants in response to both soil physical and nutritional stresses. Understanding the response patterns is critical for optimizing soil tillage and nutrient management in the field.

## Highlights

• How wheat responds to physical and nutritional stresses was investigated.
• An evident interaction between mechanical impedance and phosphorus supply were observed.
• Mechanical impedance restricted wheat growth under sufficient phosphorus supply
• Wheat responses to mechanical impedance were reduced under low phosphorus supply
• Tall and dwarf wheat genotypes performed similarly under impedance and P stresses.

## 1 Introduction

Roots are critical for the plant to acquire water and nutrients from soil. Root structure and function determine soil exploration and exploitation, and have a major impact on nutrient and water uptake, stress tolerance and crop productivity. Root structure, the spatial distribution and characteristics of root systems, are fundamentally important for the ability of plants to capture soil resources (Lynch, 2019) and sense the surrounding soil environment, sending signals to the shoots via hormone pathways (Shabala et al., 2016).

Soil physical properties, especially soil strength, profoundly affect root growth and crop yield (Correa et al., 2019). Soil strength increases rapidly as soil dries (Whalley et al., 2006). In agricultural systems, the excessive use of farm equipment or tillage at unsuitable soil water content can also result in higher soil strength (Correa et al., 2019). In the field, strong subsurface soil layers confine roots to shallower soil layers, limiting root penetration to deeper layers (Whalley et al., 2012). High soil mechanical impedance leads to root morphological modification, such as the decreased size of the root system and a lower root elongation rate (Bingham and Bengough, 2003), swollen, circular, or flattened root tips (Lipiec et al., 2012), smaller angular spread (Jin et al., 2015), and altered branching patterns depending on plant species (Potocka and Szymanowska-Pulka, 2018). In addition, increased mechanical impedance has been shown to restrict shoot performance, including decreased tiller number (Atwell, 1990; Whalley et al., 2006) and reduced leaf elongation (Coelho Filho et al., 2013; Jin et al., 2015). Previous studies showed that the leaf stunting under impeded soil was impacted by alterations in gibberellin (GA) signalling, with leaf elongation of a GA-insensitive dwarf wheat line being less affected by mechanical impedance than a GA-sensitive line (Coelho Filho et al., 2013).

Root structure and function are also influenced by soil nutrient availability. As a major low-mobility element in soil, phosphorus (P) availability plays an important role in altering root development. Studies in Arabidopsis have demonstrated that low P availability inhibits primary root growth while stimulating lateral root formation and elongation (Ruiz Herrera et al., 2015). In cereal crops such as maize (Li et al., 2012; Wang et al., 2020) and rice (Wissuwa, 2003), there is no reduction in primary root elongation in response to P deprivation. In addition, P deficiency has been shown to increase the proportion of fine roots as well as specific root length (Lyu et al., 2016; Wen et al., 2019). The reduction of shoot growth caused by low P supply has been widely demonstrated and includes reduced tiller number (Luquet et al., 2015; Rodríguez et al., 1999) as well as leaf stunting (Assuero et al., 2004; Kavanova et al., 2006). Interestingly, the GA signalling pathway is also involved in plant shoot and root responses to P starvation (Jiang et al., 2007). Inorganic phosphate (Pi) starvation down-regulates the transcript levels of GA biosynthesis genes, and causes a reduction in bioactive GA content (Jiang et al., 2007).

In the field, crops suffer a combination of physical and nutritional stresses. While the responses of crops to soil strength or P deficiency have been studied individually, little attention is given to how they interact to determine plant performance. Since both soil strength and P availability profoundly alter plant morphology, especially root development, it is important to explore the interaction between these two factors. Moreover, there is evidence that GA is involved in regulating processes in response to both mechanical impedance and P deficiency. Therefore, there could be a signaling interaction related to GA between mechanical impedance and P deficiency. Here, we tested the hypothesis that there are interaction effects between plant responses to soil mechanical impedance and P availability, and that plant responses to mechanical impedance are dependent on P availability. We investigated leaf and root growth of wheat seedlings under mechanical impedance and P availability treatments. The potential involvement of GA in these interaction processes was investigated by testing the response pattern of wheat genotypes with contrasting GA-sensitivity to mechanical impedance and P availability.

## 2 Material and methods

### 2.1 Plant material and growth condition

Two wheat (*Triticum aestivum* L.) near isogenic lines (NILs) containing *Rht-B1a* (tall allele) or *Rht-B1c* (dwarf allele) in the Cadenza background were used in this study. The *Rht-B1c* allele (from the source variety Mercia; Pearce et al., 2011) was backcrossed into cv. Cadenza with recurrent selection for the dwarfing mutation. After six rounds of backcrossing homozygous progenies were selected and bulked. Seeds were germinated between two sheets of wet filter paper in Petri dishes which were covered with aluminium foil to maintain darkness during germination. Individual germinated seeds were planted into a 2 cm deep hole in the centre of a sand column described below. Wheat seedlings were grown in a controlled environment room with a light: dark regime of 14:10 h, a temperature of 22:18 °C, humidity of 70:80 % and light intensity of 450 μmol m^−2^ s^-1^ at plant height. Plants were grown in the sand column for 40 days with or without the mechanical impedance applied from the beginning.

### 2.2 Mechanical resistance apparatus

The sand column system that was employed to investigate the effects of mechanical impedance and P availability on wheat growth is described in previous studies (Ge et al., 2019; Jin et al., 2015). Rigid plastic tubes of 45 cm in length and 15 cm in diameter were placed in tanks of nutrient solution on a base with a mesh lining. Each tank contained four tubes. The tubes were filled with sand (RH65 grade; Double Arches Quarry/Eastern Way, Leighton Buzzard LU7 9 L F, UK) together with nutrient solution to ensure sand was poured gradually and evenly into the nutrient solution. A template was used to give a sand level surface raised 8 mm above the top of the tube. The sand columns were allowed to drain to equilibrium overnight and the water table height was maintained at 30 cm below the surface of the sand. During the experiment, the roots did not reach the water table. The sand columns were then covered by a plastic disc which enabled even distribution of weight applied from above. Application of a foam weight (0.06 kg) or a steel weight (17 kg) constituted the control (CK) or impeded (IM) mechanical resistance treatment, which produced penetrometer resistance of 0.19 or 0.75 MPa, respectively (Clark et al., 2002). The foam weight and steel weight had the same shape. The porosity of the sand is approximately 30 % and it is not affected by the application of the weight, because the sand is not compressible at these confining pressures. Our previous work showed that the sand column system can precisely control the mechanical impedance independently of other properties of the growing medium, such as aeration and water status (Clark et al., 2002; Coelho Filho et al., 2013; Ge et al., 2019). When a steel weight is placed on the surface of a sand column, the mechanical impedance is increased because confining pressure makes it harder to expand cavities. However, there is negligible compressibility of the sand under the weight, and the application of the weight had a minimal effect on density (Ge et al., 2019). In this study we used sand from a geological deposit; such sands do not deform until confining pressures exceed 1000 kPa (Cheng et al., 2001). We only apply approximately 11 kPa to the sand. Even agricultural sands are relatively incompressible at these low confining pressures (see Chakraborty et al., 2014).

### 2.3 Nutrient solutions

Two levels of P treatment were applied. P was included as either 250 or 10 μM KH2PO4 in the Hoagland solution in high P (HP) or low P (LP) treatments, respectively. To maintain an equimolar K concentration, KCl was added to the LP treatment. The nutrient solution composition apart from P was 2 mM Ca(NO_3_)_2_, 0.75 mM K_2_SO_4_, 0.65 mM MgSO_4_, 0.1 mM KCl, 1.0 × 10^−3^ mM H_3_BO_3_, 1.0 × 10^−3^ mM MnSO_4_, 1.0 × 10^−4^ mM CuSO_4_, 1.0 × 10^−3^ mM ZnSO_4_, 5.0 × 10^−6^ mM (NH_4_)_6_Mo_7_O_24_ and 0.1 mM Fe-EDTA. The pH of the solution was adjusted to 6.0. A final volume of approximately 80 L of nutrient solution was supplied in each tank, and the nutrient solution in the tanks was replaced 20 days after the start of the experiment.

### 2.4 Plant measurements

During the experiment, the length of the leaf blade on the first tiller was measured daily with a Perspex ruler. At harvest the number of tillers and nodal roots were counted and the length of the longest leaf was measured. Roots were washed free of sand, and shoot and root samples were collected separately. Leaf blades were scanned at a resolution of 400 dpi immediately after harvesting. Fresh roots were scanned at a resolution of 400 dpi. Leaf and root images were analysed using WinRhizo (Regent Instruments, Quebec, Canada) to obtain leaf area, total root length, number of root tips, and root diameter. Nodal roots and embryonic roots were analysed separately. The axial length and lateral root length of nodal roots were measured on scanned images using Image J software (Version 1.4, http://rsb.info.nih.gov/ij). The root branching intensity was determined by dividing the number of root tips by the total root length. Root diameters (d) were recorded in 31 classes between 0 and 3.0 mm, which were bulked into 5 groups: 0 < d ≤ 0.2, 0.2 < d ≤ 0.4, 0.4 < d ≤ 1, 1.0 < d ≤ 2.0, and d >2.0 mm. After scanning, shoot and root samples were oven dried at 70 °C to a constant weight to measure the dry weight. The oven-dried material was ground to a powder and digested using a mixture of nitric acid and perchloric acid (85:15 V/V) in open tube digestion blocks. The acids are removed by volatilisation and the residue dissolved in nitric acid (5 % V/V). The solution was used to measure P content with inductively coupled plasma optical-emission spectroscopy (ICP-OES, OPTIMA 3300 DV, Perkin-Elmer, Waltham, MA, USA).

### 2.5 Experimental treatments and statistical analysis

There were three treatment factors in the present study: two wheat genotypes (*Rht-B1a* and *Rht-B1c*), two levels of mechanical resistance (CK and IM), and two P levels (HP and LP), to give eight treatment combinations with 4 replicates for each treatment. The experiment was arranged with randomized complete block design. Every block consisted of two tanks (high P or low P) to avoid contamination with P. Each tank contained six experimental units, which represented three wheat genotypes under two levels of mechanical impedance (the third genotype is not discussed in this paper). Analysis of Variance (ANOVA) with the block factor and post-hoc Tukey HSD test at the 5 % probability level was used to determine differences among treatments. Statistical analysis of the leaf elongation measurements was done by modelling the general response as a linear regression and then superimposing the approximate sigmoid shape over time using splines, all in the context of Residual Maximum Likelihood (REML, Jin et al., 2015). Principal component analysis (PCA) among shoot or root traits of wheat genotypes in response to mechanical impedance and P stress was performed, using the ‘vegan’ package. Shoot biomass, leaf area, tiller number, and length of the longest leaf were used in shoot traits PCA; root biomass, total root length, nodal root number, specific root length, axial length of nodal roots, lateral root length, and root branching intensity were used in root traits PCA. The statistical analyses were conducted with R version 3.5.0 (R Development Core Team, 2018).

## 3 Results

### 3.1 Biomass and P uptake

**Fig. 1:**
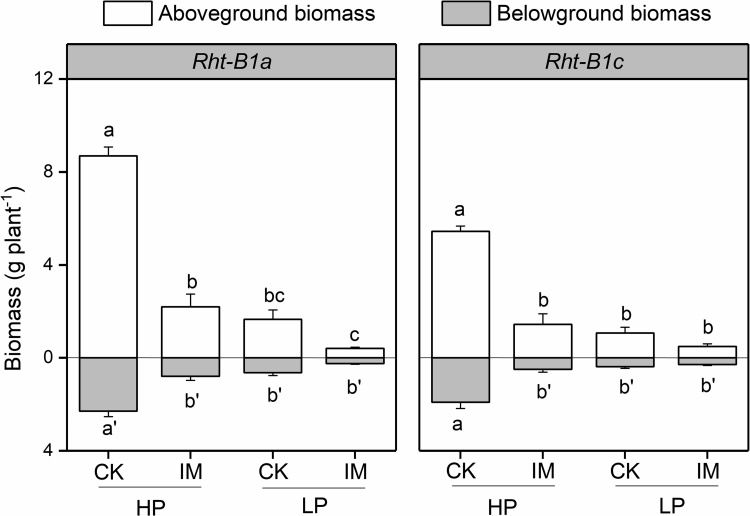
The effect of mechanical impedance and phosphorus supply on the aboveground (white bars) and belowground (grey bars) biomass of two wheat genotypes at harvest. Bars indicate means + SE (n = 4 individual plants). Different letters indicate significant differences among treatments on each wheat genotype (*P* < 0.05). CK: low impedance control check; IM: impeded plants; HP: high phosphorus; LP: low phosphorus.

The effect of mechanical impedance on wheat growth and morphology was determined by growing plants in the sand column system with contrasting P supply. Three-way ANOVA showed that the main effects of mechanical impedance and P supply, as well as their interaction effect, on shoot and root biomass were significant at P < 0.001 (Table 1). Mechanical impedance significantly reduced plant shoot and root biomass under high P (HP) supply in both wheat genotypes, *Rht-B1a* and *Rht-B1c* (Fig. 1). Under HP, the shoot and root biomass in impeded (IM) *Rht-B1a* plants was 75 % and 66 %, respectively, lower than those in the low impedance control (CK). While under low P (LP) supply, the shoot and root biomass in control (CK) or impeded (IM) plants showed no significant differences for both *Rht-B1a* and *Rht-B1c* (Fig. 1). The main effect of wheat genotype on shoot biomass was significant at P < 0.001, while the effect on root biomass was not significant (Table 1). The shoot biomass of *Rht-B1a* plants was higher than *Rht-B1c*, while the root biomass was similar.

**Table 1:** The effect of mechanical impedance and P supply on shoot and root traits in two wheat genotypes at the point of harvest. Three-way ANOVA was conducted. F value for wheat genotype, mechanical impedance, P levels, and their interaction were reported. Note: ns: no significant differences; *: *P* < 0.05, **: *P* < 0.01, ***: *P* < 0.001.

	Shoot biomass	Tiller number	Leaf area	Length of the longest leaf	Root biomass	Total root length	Nodal root number	Root branching intensity	Axial root length	Lateral root length	Plant P content
Block	0.04^ns^	0.61^ns^	0.26^ns^	1.04^ns^	0.58^ns^	0.43^ns^	0.47^ns^	1.84^ns^	0.05^ns^	0.56^ns^	0.14^ns^
Genotype (G)	18.97***	0.23^ns^	9.66**	122.29***	3.98^ns^	4.42*	2.67^ns^	1.67^ns^	0.11^ns^	2.16^ns^	5.49^ns^
Impedance (IM)	141.48***	84.28***	121.93***	22.55***	55.82***	193.09***	182.39***	66.8***	97.76***	56.91***	141.79***
Phosphorus (P)	186.49***	59.05***	107.3***	42.35***	74.78***	68.46***	230.65***	23.51***	2.82^ns^	0^ns^	198.9***
G * IM	9.44**	0^ns^	5.47^ns^	4.26^ns^	0.65^ns^	3.7^ns^	1.53^ns^	0.52^ns^	0.81^ns^	0.86^ns^	3.21^ns^
G * P	11.4**	1.67^ns^	1.4^ns^	8.01*	1.03^ns^	2.68^ns^	3.72^ns^	4.01^ns^	0.32^ns^	1.5^ns^	4.88*
IM * P	69.84***	18.35***	29.68***	9.16**	28.61***	41.68***	85.58***	11.94***	2.39^ns^	1.21^ns^	75.37***
G * IM * P	3.08^ns^	0.12^ns^	0.03^ns^	0.76^ns^	0.23^ns^	0.01^ns^	1.29^ns^	4.4*	0.63^ns^	2.29^ns^	0.64^ns^

### 3.2 Shoot morphology

**Fig. 2:**
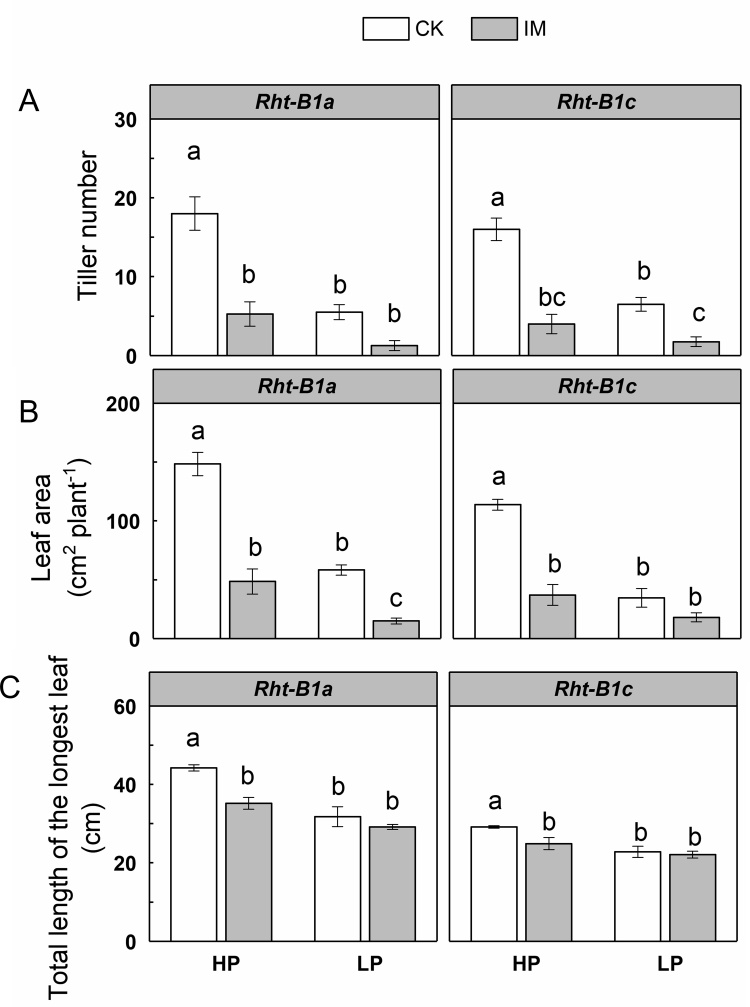
The effect of mechanical impedance and phosphorus supply on the tiller number (A), leaf area (B), and the total length of the longest leaf (C) of two wheat genotypes at harvest. Bars indicate means ± SE (n = 4 individual plants). Different letters indicate significant differences among treatments on each wheat genotype (*P* < 0.05). The white bars show data for plants growing in the low impedance control, the grey bars show data for the plants under mechanical impedance. HP: high P supply; LP: low P supply.

**Fig. 3:**
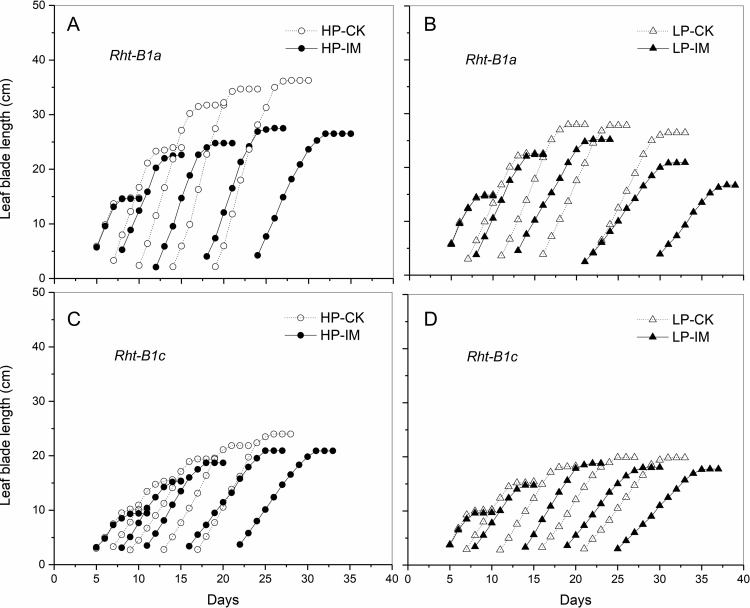
The effect of mechanical impedance and phosphorus supply on the leaf blade elongation (leaf 1 up to 5) of two wheat genotypes. The open symbols represent low impedance control (CK); the filled symbols represent the mechanically impeded treatments (IM). The left panels (A and C) show the leaf blade elongation in response to mechanical impedance under high P (HP) condition; the right panels (B and D) show leaf blade elongation under low P condition. The plots show means of leaf blade lengths from 4 individual plants. For *Rht-B1a*, the main effects of mechanical impedance and P level and the interaction effect were significant at *P* < 0.001. For *Rht-B1c*, the main effect of P level was significant at *P* = 0.04; the main effect of mechanical impedance was not significant.

There were significant interaction effects between mechanical impedance and P level on tiller number, leaf area, and length of the longest leaf (P < 0.001, Table 1). The leaf area, and the longest leaf length of *Rht-B1a* were greater than *Rht-B1c* in all treatments, but the tiller number was not affected by genotype (Table1). The number of tillers was greatly reduced (71 %) by mechanical impedance compared to CK treatment under HP supply, while there was no significant change in tiller number between CK and IM plants under LP supply in *Rht-B1a* (Fig. 2A). In *Rht-B1c*, IM plants showed a significant decrease in tiller number in comparison to CK plants under both HP and LP supply. Leaf area of IM plants was significantly smaller than CK plants under HP supply in both genotypes (Fig. 2B). Under LP supply, IM reduced the leaf area in *Rht-B1a*, but not in *Rht-B1c*. Mechanically impeded plants had a lower length of the longest leaf compared with the low impedance control plants under HP supply, while mechanical impedance did not affect the longest leaf length under LP supply, for both *Rht-B1a* and *Rht-B1c* (Fig. 2C). The length of the longest leaf of IM plants was 20 % lower than for the CK plants under HP supply in *Rht-B1a.* In comparison with *Rht-B1a*, the effect of mechanical impedance on length of the longest leaf was relatively small in *Rht-B1c*, with only a 13.5 % reduction being observed. The effect of mechanical impedance on leaf elongation under contrasting P supply is shown in Fig. 3. In all cases impedance delayed leaf emergence (Fig. 3). Elongation of the leaf blade was stunted by mechanical impedance under HP supply (Fig. 3A), while the stunting effect of IM was much smaller in the first three leaves under LP supply in *Rht-B1a* (Fig. 3B). In *Rht-B1a*, the blade length of the third leaf of IM plants was 22 % less than of CK plants under HP supply, while it was only 9 % less than CK plants under LP supply. The main effect of IM on leaf elongation in *Rht-B1c* was not significant (Fig. 3C, D).

### 3.3 Root morphology

**Fig. 4:**
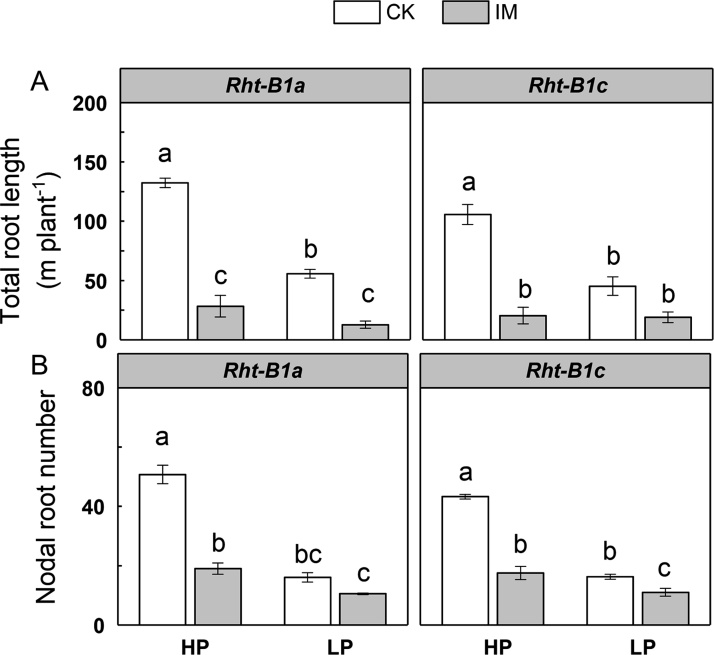
The effect of mechanical impedance and phosphorus supply on the total root length (A) and nodal root number (B) of two wheat genotypes at harvest. Bars indicate means ± SE (n = 4 individual plants). Different letters indicate significant differences among treatments on each wheat genotype (*P* < 0.05). For explanation of the treatments, see Fig. 2.

**Fig. 5:**
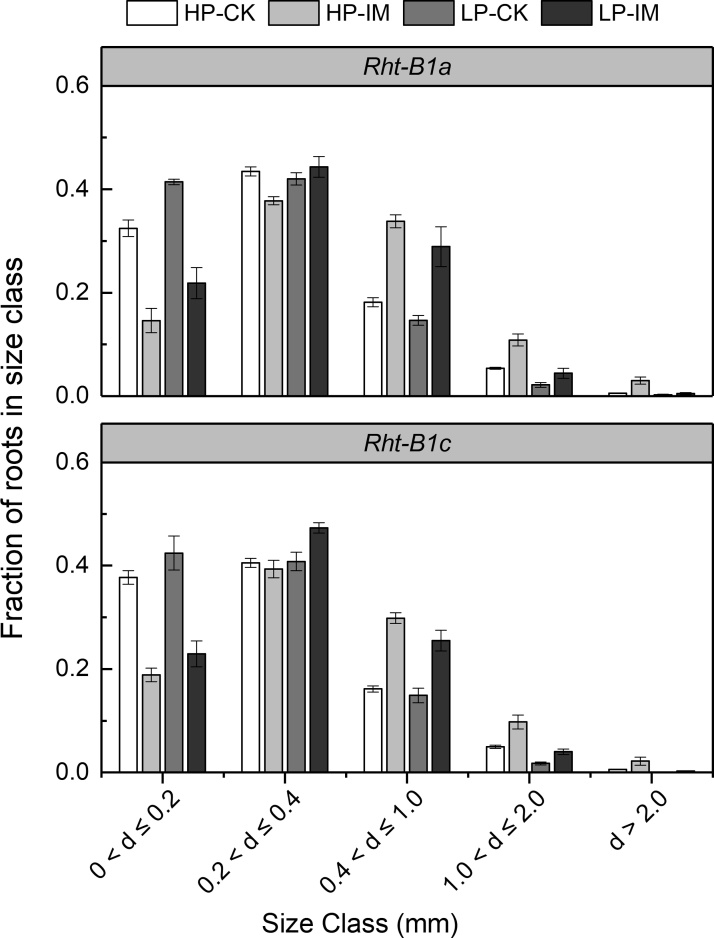
The effect of mechanical impedance and phosphorus supply on root diameter size distribution of two wheat genotypes at harvest. Bars indicate means ± SE (n = 4 individual plants). For both wheat near isogenic lines (NILs), the main effects of mechanical impedance and P level and the interaction effect were significant at *P* < 0.001. HP: high P supply; LP: low P supply; CK: low impedance control; IM: mechanical impedance.

**Fig. 6:**
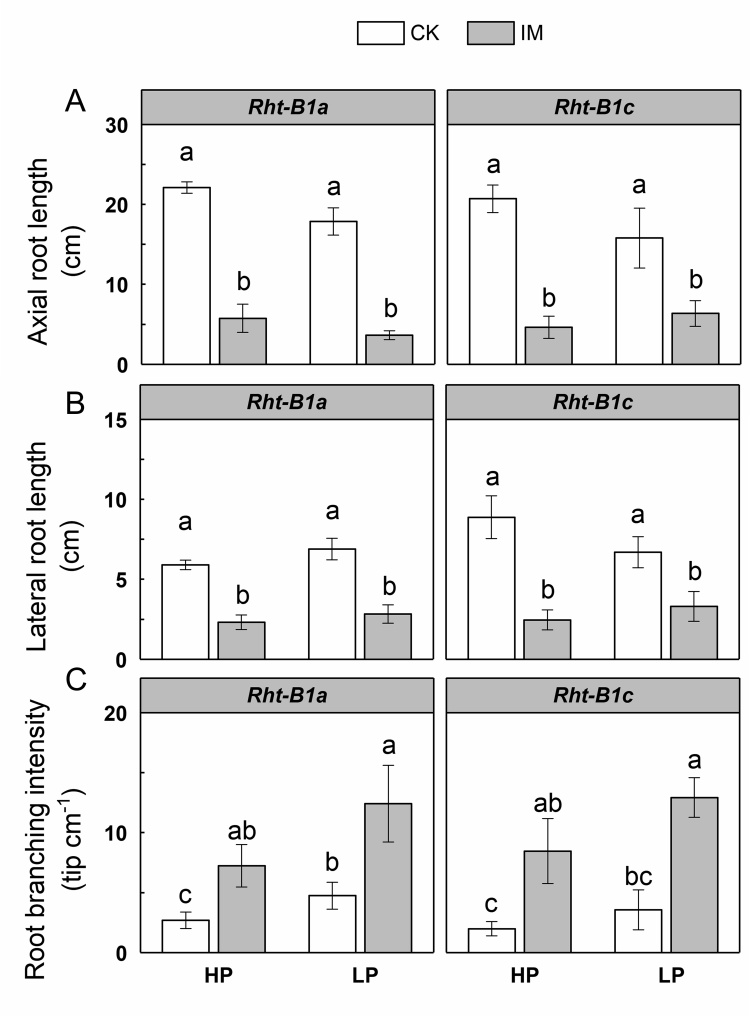
The effect of mechanical impedance and phosphorus supply on axial root length (A), lateral root length (B), and root branching intensity (C) of two wheat genotypes at harvest. Bars indicate means ± SE (n = 4 individual plants). Different letters indicate significant differences among treatments on each wheat genotype (*P* < 0.05). For explanation of the treatments, see Fig. 2.

**Fig. 7:**
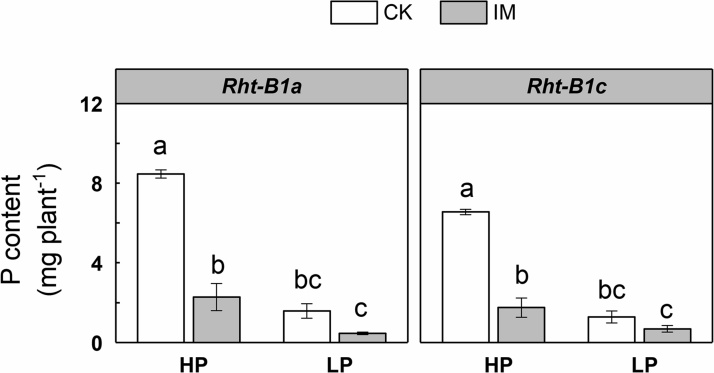
The effect of mechanical impedance and P supply on the P uptake of two wheat genotypes at harvest. Bars indicate means ± SE (n = 4 individual plants). Different letters indicate significant differences among treatments on each wheat genotype (*P* < 0.05). For explanation of the treatments, see Fig. 2.

The main effects of mechanical impedance, P level, as well as their interaction, on total root length, nodal root number, and root branching intensity were significant at *P* < 0.001 (Table 1). However, the main effect of P level and the interaction effect between P and impedance on axial root length and lateral root length were not significant (Table 1). Wheat genotype had no significant effect on branching intensity, nodal root number, axial length of nodal root, and lateral root length (Table 1). Mechanically impeded plants showed lower total root length compared to low impedance control plants under both HP and LP supply in *Rht-B1a* (Fig. 4A). In *Rht-B1a*, the total root length of IM plants was 79 % and 78 % less than the CK plants under HP and LP supply, respectively. In *Rht-B1c*, total root length was decreased 81 % by IM under HP supply, while there was no significant difference between CK and IM total root length under LP supply (Fig. 4A). Wheat genotype had a significant individual effect (with no interactions with IM or P) on total root length (Table 1). *Rht-B1a* plants had greater total root length than *Rht-B1c,* independent of mechanical impedance or P supply (Fig. 4A, Table 1). IM plants showed fewer nodal roots than CK plants under HP in both genotypes, while the effect under LP was much smaller (Fig. 4B). The distribution of root diameters for plants of each treatment is shown in Fig. 5. Roots were thicker under mechanical impedance, which resulted in a reduction in fine roots (0 < d ≤ 0.2 mm) and an increase in thicker roots (0.4 < d ≤ 1.0 mm) under both P levels. Under LP supply, impeded plants did not show a significantly increased proportion of root diameters larger than 1.0 mm (d > 1.0) compared to low impedance control. Low P supply increased the proportion of fine roots under low mechanical impedance. ANOVA showed the main effects of mechanical impedance and P level, as well as their interaction on root diameter were significant at P < 0.001. Mechanical impedance also restricted wheat root elongation (Fig. 6A, B). The axial length of nodal roots and the lateral root length were greatly reduced by mechanical impedance under both HP and LP in both genotypes. Mechanical impedance also increased root branching intensity in both HP and LP in both genotypes (Fig. 6C). In addition, root tip deformation was observed in the mechanically impeded plants under both HP and LP supply (data not shown). Mechanical impedance and low P supply caused a reduction in plant P content (Fig. 7). Plants under LP supply showed lower P content compared to plants under HP supply. Under HP supply, IM plants showed a 73 % lower P content in comparison to CK plants in both *Rht-B1a* and *Rht-B1c*, while mechanical impedance did not significantly affect P content under LP supply (Fig. 7).

### 3.4 Interaction effects

**Fig. 8:**
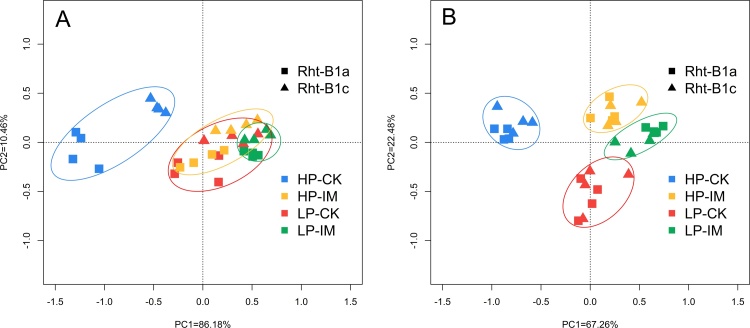
Principal component analysis (PCA) of shoot (A) and root (B) traits among treatments and wheat genotypes. PC1 represents the first axis, PC2 represents the second axis, and the percentage number represents proportion of variation the axis could explain. Shoot biomass, leaf area, tiller number, and length of the longest leaf were used in shoot traits PCA; root biomass, total root length, nodal root number, specific root length, axial length of nodal roots, lateral root length, and root branching intensity were used in root traits PCA. For explanation of the treatments, see Fig. 2.

Principle component analysis (PCA) was performed to show the interaction effect between mechanical impedance and P level on shoot and root traits in both wheat genotypes (Fig. 8). For shoot traits (Fig. 8A), shoot biomass, leaf area, tiller number, and the total length of the longest leaf were used in PCA. PC1 separated HP-CK treatment from the other three treatments. HP-IM, LP-CK, and LP-IM had a similar shoot traits pattern. In addition, the two wheat genotypes were separated in the HP-CK treatment but not in the other three treatments. For root traits (Fig. 8B), root biomass, total root length, nodal root number, specific root length, axial length of nodal roots, lateral root length, and root branching intensity were used in PCA. PC1 separated HP-CK from HP-IM, while LP-CK and LP-IM were relatively close. The two wheat genotypes were not separated in any of the treatments.

## 4 Discussion

### 4.1 Effects of mechanical impedance under sufficient P supply

Mechanical impedance applies strong shear and compressive force to root penetration, greatly affecting root growth. Our results showed that mechanical impedance significantly restricted root growth and development (Fig. 4, Fig. 5, Fig. 6), which is consistent with previous studies (Alameda et al., 2012; Bingham and Bengough, 2003; Lipiec et al., 2012). The root system of wheat is composed of two root types, the embryonic seminal roots and adventitious nodal roots (Klepper et al., 1984). The number of seminal root axes is about 3–6, determined by the genotype, while the number of nodal roots is very plastic and largely governed by the environment (Eshel and Beeckman, 2013). In the present study, mechanical impedance caused a significant reduction in nodal root number, which corresponds to previous studies in wheat (Colombi and Walter, 2017; Jin et al., 2015). Root diameter was increased under mechanical impedance (Fig. 5) as shown in a number of studies (Pfeifer et al., 2014; Potocka and Szymanowska-Pulka, 2018; Tracy et al., 2011). Increased root diameter could be an adaptive strategy in response to mechanical impedance. Thicker roots lead to greater axial growth force, providing an improved penetration ability in strong soil (Bengough et al., 2011) and possibly also increased surface area for nutrient uptake. In addition, our results showed that nodal roots of impeded plants had a shorter axial length (Fig. 6A), suggesting that mechanical impedance restricted root axial penetration to deeper soil. The lateral root length was also reduced by mechanical impedance. Interestingly, the effect of impedance on the elongation of lateral roots was much smaller than that on axial roots. The impeded axial root length was 22 % of the control, while the lateral root length was 40 % of the control in *Rht-B1a* under HP supply (Fig. 6A, B), implying axial root elongation was more sensitive than lateral root elongation. Moreover, our results showed that the root branching intensity was increased under IM (Fig. 6C). Similarly, several studies showed mechanical impedance has a stronger effect on axial root than lateral root elongation, and the reduction of axial elongation rate is accompanied by an increase in branching intensity (Bingham and Bengough, 2003; Thaler and Pagès, 1999). The reason could be related to the compensatory adjustments of lateral roots when the main axial roots were significantly restricted (Bingham and Bengough, 2003; Kolb et al., 2017). How roots sense mechanical impedance remains uncertain. There is some evidence for an increase in the turgor pressure of growing root cells in response to mechanical impedance (Goss and Russell, 1980; Kolb et al., 2017), but the mechanism still needs further investigation. Root length, especially that of fine roots, determines the ability to explore the soil, which is critical for plant P acquisition (Wen et al., 2019). Root tips also play an important role in the total seedling P uptake despite their small size (Kanno et al., 2016). Impedance-induced reduction in root exploration and root tip deformation leads to a significant decrease in P uptake in impeded plants (Fig. 7).

In the present study, wheat shoot biomass and development were significantly reduced by mechanical impedance when nutrient supply was sufficient (Fig. 1, Fig. 2, Fig. 3). Decreased tiller number, leaf area and elongation were observed in impeded plants, which is consistent with previous studies (Coelho Filho et al., 2013; Jin et al., 2015). Some shoot and root traits, such as nodal root number and the longest leaf length, showed a similar response pattern to mechanical impedance. The co-ordination of growth between wheat shoot and root has been shown in several papers. Nodal root number is positively correlated with plant height (Colombi and Walter, 2017), leaf number (Klepper et al., 1984), and tiller number (Ge et al., 2019), and total root length shows strong correlation with leaf area (Jin et al., 2015). The restricted shoot growth could be related to the reduced P uptake in the impeded plants. Hormonal signaling also plays an important role in triggering the initial plant responses to mechanical impedance (Masle and Passioura, 1987). For example, ethylene (Sarquis et al., 1991) and GA (Coelho Filho et al., 2013) have been shown to be involved in shoot architecture alteration under mechanical impedance. However, the detailed role of phytohormones in mediating plant growth in response to mechanical impedance needs more extensive investigation.

### 4.2 P levels shape plant responses under mechanical impedance

Our results suggest a strong interaction between mechanical impedance and P supply level. Three-way ANOVA results showed the significant interaction effects between IM and P on a series of plant traits, including shoot and root biomass, tiller number, leaf area, length of the longest leaf, root biomass, total root length, nodal root number, root branching intensity, and plant P content (Table 1). Under HP supply, mechanical impedance significantly restricted shoot and root growth, while under LP supply, impeded plants showed a similar performance to the low impedance control (Fig. 1). In the present study, we dissected the potential interaction effect between mechanical impedance and P availability with the sand column system which provides a precise control of physical aspects of the root environment and allows mechanical impedance to be isolated from water availability and solute transport (Clark et al., 2002). Indeed, the difference in P acquisition between impeded plants and the low impedance control was smaller under LP supply (Fig. 7), explaining part of the interaction effect. PCA plots showed different patterns of the interaction effects on shoot and root traits (Fig. 8), implying the interaction cannot be explained by differences in nutrient acquisition alone. Moreover, the two genotypes with contrasting GA sensitivity performed similarly in response to impedance and P stresses, implying GA sensitivity may not be the main mechanism underlying the interaction between IM and P. In the present study, leaf elongation was reduced by mechanical impedance in both genotypes and P levels (Fig. 3). This reduction in leaf elongation caused by IM was more pronounced with plant age, which may be related to nutrient limitation as a result of restricted rooting and lower exploration under IM, especially when plants get larger and need more nutrients. It is noteworthy that the leaf blade stunting in response to IM in the third leaf was less under LP in the tall genotype (Fig. 3A, B), which could not be explained by the nutrient effect alone, but may be mediated by the interaction between P and IM. Root formation and branching processes (nodal root number and root branching intensity) were significantly affected by the interaction between IM and P, while the interaction effect on root elongation (axial and lateral root length) was not significant, suggesting the interaction was related to a specific regulation process. Previous studies showed that both mechanical impedance and low phosphorus have significant impacts on the whole root system architecture (RSA, Correa et al., 2019; Lynch, 2019). Impeded roots can grow more steeply than non-impeded control (Jin et al., 2015). Under P limitation, plants tend to convert to a topsoil foraging root system, including shallower growth angles of axial roots, enhanced adventitious rooting, and greater branching of lateral roots (Lynch, 2011). In the present study, the axial length of nodal roots and the lateral root length were greatly reduced by mechanical impedance under both HP and LP conditions (Fig. 6A, B). Further study of rooting depth and spread angle of roots would be helpful to understand the possible interaction between IM and LP on the overall RSA. A study of the interaction between soil compaction and nitrogen (N) showed that there was no significant interaction between compaction and N supply on plant growth and biomass partitioning (Bingham et al., 2010). Our previous finding with the same sand culture system suggested that leaf stunting caused by mechanical impedance was irrespective of N availability (Ge et al., 2019). Comparing with these above studies, our results indicated a novel interaction between mechanical impedance and P availability, which could be related to a signaling interaction rather than a nutritional deprivation-triggered process.

### 4.3 Wheat genotype and the possible GA involvement

Our results suggested a potential involvement of GA sensitivity in plant response to mechanical impedance and P stress. In the present study, two wheat NILs containing tall or dwarfing *Rht-1* alleles with contrasting sensitivity to GA were used to test their performance under mechanical and P stresses. Shoot biomass, leaf area, and leaf elongation were significantly influenced by wheat genotype. *Rht-B1c* was more tolerant of mechanical impedance and P stress in terms of shoot biomass (Fig. 1). We found that leaf stunting in response to mechanical impedance in the GA sensitive genotype *Rht-B1a* was much stronger than that in the GA-insensitive genotype *Rht-B1c*, which is consistent with a previous study (Coelho Filho et al., 2013). Besides, the PCA showed that the two wheat genotypes were separated only in shoot traits under the HP-CK treatment, indicating the differences between these two genotypes are not apparent under mechanical impedance and P stress.

## 5 Conclusions

Mechanical impedance reduced wheat shoot and root growth under sufficient P supply, whereas under low P supply the effects of mechanical impedance on wheat growth were restricted. Shoot and root biomass, tiller number, leaf elongation, and nodal root number were significantly decreased in impeded plants under HP supply, but not under LP supply, suggesting that wheat growth restriction in response to mechanical impedance is dependent on P supply. Two wheat genotypes with contrasting GA sensitivity performed similarly under combined impedance and P stresses. These findings providing new insights into the integrated adaptation of plants to both soil physical and nutritional stresses, implying the need to consider coupling of soil physical and nutritional management in agricultural practice.

## Declaration of Competing Interest

There are no conflicts of interest to disclose.
